# Feasibility and Accuracy of Sentinel Lymph Node Biopsy in Clinically Node-Positive Breast Cancer after Neoadjuvant Chemotherapy: A Meta-Analysis

**DOI:** 10.1371/journal.pone.0105316

**Published:** 2014-09-11

**Authors:** Jian-Fei Fu, Hai-Long Chen, Jiao Yang, Cheng-Hao Yi, Shu Zheng

**Affiliations:** 1 Key Laboratory of Cancer Prevention and Intervention, Chinese Ministry of Education, Key Laboratory of Molecular Biology in Medical Sciences, Hangzhou, Zhejiang Province, China; 2 Institute of Oncology, The Second Affiliated Hospital of Zhejiang University School of Medicine, Hangzhou, Zhejiang Province, China; 3 Department of Oncology, Jinhua Central Hospital, Jinhua Hospital of Zhejiang University School of Medicine, Jinhua City, Zhejiang Province, China; University of North Carolina School of Medicine, United States of America

## Abstract

Sentinel lymph node biopsy (SLNB) has replaced conventional axillary lymph node dissection (ALND) in axillary node-negative breast cancer patients. However, the use of SLNB remains controversial in patients after neoadjuvant chemotherapy (NAC). The aim of this review is to evaluate the feasibility and accuracy of SLNB after NAC in clinically node-positive patients. Systematic searches were performed in the PubMed, Embase, and Cochrane Library databases from 1993 to December 2013 for studies on node-positive breast cancer patients who underwent SLNB after NAC followed by ALND. Of 436 identified studies, 15 were included in this review, with a total of 2,471 patients. The pooled identification rate (IR) of SLNB was 89% [95% confidence interval (CI) 85–93%], and the false negative rate (FNR) of SLNB was 14% (95% CI 10–17%). The heterogeneity of FNR was analyzed by meta-regression, and the results revealed that immunohistochemistry (IHC) staining may represent an independent factor (*P* = 0.04). FNR was lower in the IHC combined with hematoxylin and eosin (H&E) staining subgroup than in the H&E staining alone subgroup, with values of 8.7% versus 16.0%, respectively (*P* = 0.001). Thus, SLNB was feasible after NAC in node-positive breast cancer patients. In addition, the IR of SLNB was respectable, although the FNR of SLNB was poor and requires further improvement. These findings indicate that IHC may improve the accuracy of SLNB.

## Introduction

The presence of axillary lymph node metastases, as one of the strongest predictors of survival, is necessary for accurate staging and the selection of local and systemic adjuvant therapies [1]–[3]. The status of axillary lymph nodes can be confirmed by complete axillary lymph node dissection (ALND), which will cause morbidities in nearly 20% of patients, such as lymphedema of the upper limb, tenderness, and movement disorders of the shoulder girdle[4]. In clinically node-negative patients, sentinel lymph node biopsy (SLNB), as a minimally invasive staging tool, can predict the status of axillary lymph nodes with an identification rate (IR) of more than 90% and a false negative rate (FNR) of less than 10%[5], [6]. The clinical trials of ACOSOG Z0010 and Z0011 indicated that the use of SLNB for staging axillary lymph nodes exhibited a similar relapse rate in comparison with ALND[7], [8]. For clinically node-negative patients, SLNB has replaced ALND as the standard procedure to address axillary lymph node status. In recent years, neoadjuvant chemotherapy (NAC) has played an increasingly important role in the comprehensive treatment of locally advanced breast cancer[9], [10]. NAC is frequently recommended for node-positive patients, of which 40% can achieve pathologically complete response of their axillary nodes[10].

Many factors can impact the feasibility and accuracy of SLNB after NAC, including the status of the axillary lymph nodes, and it is therefore necessary to know whether SLNB after NAC for node-positive breast cancer patients is accurate or not. The crucial issue is whether SLNB for such patients can achieve outcomes comparable to those in clinically node-negative patients without chemotherapy. The patient selection criteria as well as the technique of mapping and detecting the metastasis of sentinel lymph nodes vary across individual studies; thus, it is difficult to determine individual patient approaches in clinical practice. This systemic review attempts to collect data for evaluation.

## Methods

### 2.1 Literature search strategy

The electronic databases PubMed (Medline), Embase, and the Cochrane Library were searched from 1993 to December 2013. The year 1993 was selected because the first publication on SLNB was published in this year. The following free text terms and medical subject heading (Mesh) terms were used: (“breast cancer” OR “breast neoplasm”) AND (“SLNB” OR “sentinel lymph node biopsy” OR “sentinel lymph node dissection’) AND (“preoperative therapy” OR “preoperative chemotherapy” OR “neoadjuvant chemotherapy”). Only articles published in English were selected. Two reviewers independently evaluated the titles and abstracts of the identified articles. Potentially relevant articles were retrieved to review the full text.

### 2.2 Study inclusion criteria

The inclusion criteria were as follows: breast cancer patients diagnosed with metastasis of the axillary lymph node by physical examination or ultrasonic image, with or without fine needle aspiration (FNA) or core needle biopsy; patients scheduled to receive NAC; and patients undergoing SLNB after NAC, followed by ALND. The exclusion criteria were as follows: patients receiving neoadjuvant endocrine therapy or preoperative radiotherapy and patients diagnosed with inflammatory breast cancer.

### 2.3 Study quality assessment

QUADAS 2 was adapted in our review[11]. It is comprised of four key domains: patient selection, index test, reference standard, and flow and timing. Each domain is assessed in terms of risk of bias and the first three are also assessed on Applicability concerns. Signalling questions are included to assist in judgements about the risk of bias. Risk of bias was judged as ‘low risk’ if the answers to all signaling questions for a domain were ‘yes’, as ‘high risk’ if any signaling question in a domain was ‘no’, or as ‘unclear risk’ when insufficient data were provided to make a judgement. Applicability concerns were judged as low risk, high risk or unclear risk with similar criteria. All studies were analyzed by two reviewers independently and any disagreement was resolved by consensus. The signaling questions adopted in our review are provided in File S1.

### 2.4 Data extraction

IR was defined as the number of patients in whom sentinel lymph nodes were successfully identified divided by the total number of patients in whom SLNB was attempted. The histological analysis of nodes collected from ALND was taken as the “gold standard”. Patients in whom sentinel lymph nodes were successfully identified were further categorized as true positive (TP), true negative (TN), or false negative (FN). A 2×2 contingency table was constructed to determine the FNR, negative predictive value (NPV), and accuracy of SLNB after NAC. FNR was equal to FN/(FN+TP), and NPV was defined as TN/(TN+FN). The accuracy was defined as (TN+TP)/(TN+FN+TP). The results of ALND are positive in sentinel node-positive patients, and SLNB exhibits no false-positive results; therefore, the specificity and positive predictive value were not considered.

### 2.5 Statistical analysis

Meta-analyses of IR and accuracy were calculated using a random effects model. The Midas module of Stata software was selected to generate pooled outcomes of FNR and NPV. Meta-regression was used to analyze the heterogeneity of different variables. The extent of heterogeneity among studies was evaluated using the inconsistency statistic (I^2^). Publication bias was detected by constructing a Funnel plot. Begg's test was used to quantitatively confirm the results of the Funnel plot. The use of immunohistochemistry (IHC) on the FNR was performed using the chi-squared test. Statistical significance was considered at *P*<0.05. All analyses were performed using Stata version 12.0.

## Results

### 3.1 The quality of the literature studies

Bias risk was based on four domains: patient selection, test index, reference index, and flow and timing. Applicability was based on three domains: patient selection, test index, and reference. A total of seven parameters were used to assess study quality. In the included studies, only risk of bias in domain of patient selection was high risk, and the other aspects were assessed as low risk. The qualities of included studies were moderate to high based on scoring using QUADAS-2 system. The results of the quality assessment are listed in Table 1.

**Table 1:** The results of quality assessment according to QUADAS 2 for the included studies.

Study	Risk of bias				Applicability concerns		
	Patient selection	Index test	Reference standard	Flow and timing	Patient selection	Index test	Reference standard
Alvarado et al.[12]	2	1	1	1	1	1	1
Boughey et al.[13]	2	1	1	1	1	1	1
Brown et al.[14]	2	1	1	1	1	1	1
Canavese et al.[15]	1	1	1	1	2	1	1
Classe et al.[16]	2	1	1	2	2	2	1
Kuehn et al.[17]	2	1	1	2	2	2	1
Lee et al.[18]	2	1	1	2	1	1	1
Newman et al.[19]	2	1	1	1	1	1	1
Ozmen et al.[20]	2	1	1	1	1	1	1
Park et al.[21]	2	1	1	1	1	1	1
Rebollo-Aguirre et al.[22]	2	1	1	?	1	2	1
Shen et al.[23]	2	1	1	1	1	2	1
Takei et al.[24]	2	1	1	2	2	2	1
Thomas et al.[25]	2	1	1	2	1	2	1
Yagata et al.[26]	2	1	1	2	1	1	1

1: low risk. 2: high risk?: unclear risk.

### 3.2 Characteristics of the studies identified

**Figure 1:**
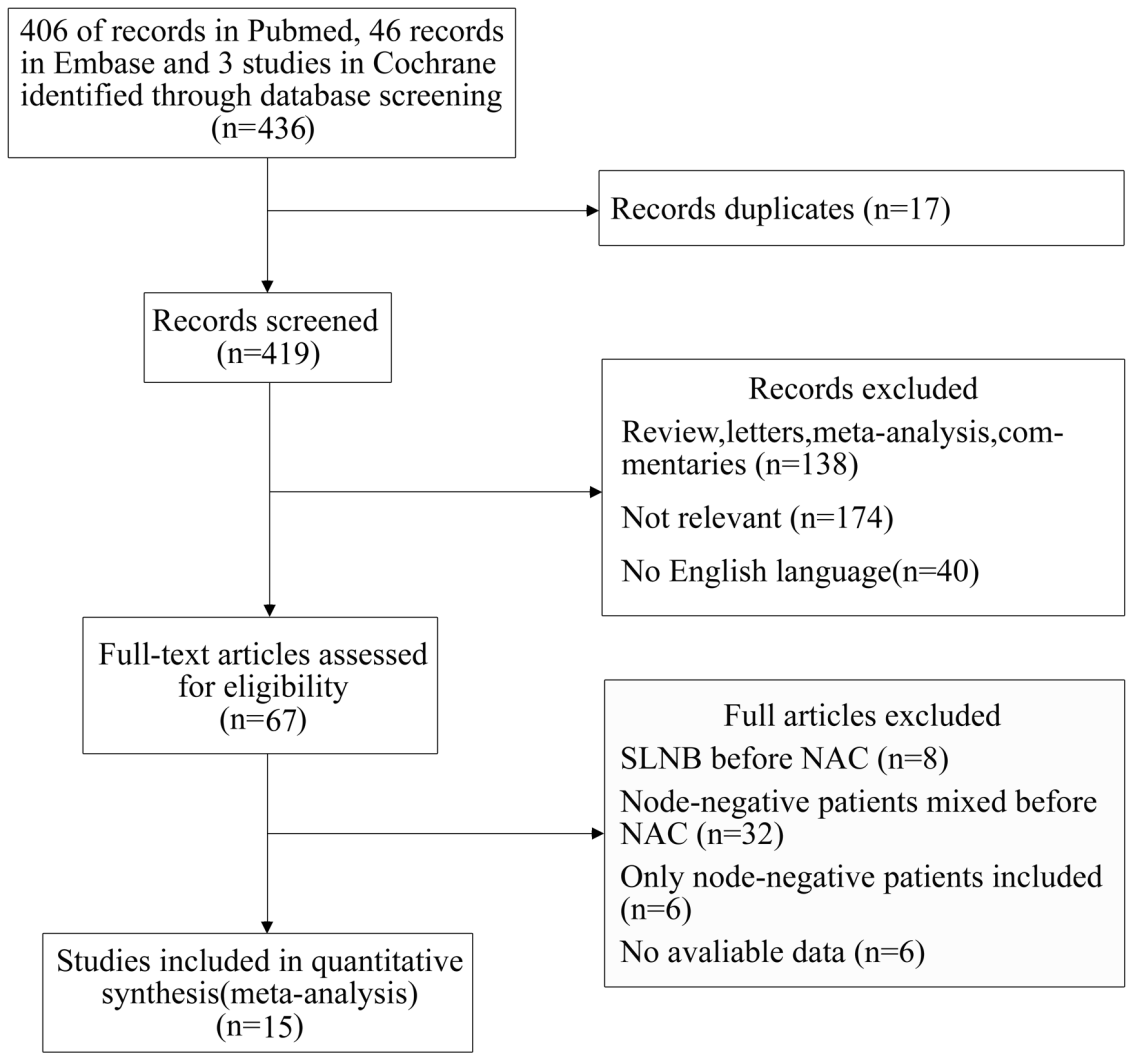
Study screening process: Flow diagram.

Of 436 eligible studies, 369 articles were excluded due to duplicates, reviews, letters, meta-analysis, and commentaries. Sixty-seven full-length articles were retrieved; of these, 32 articles were excluded because of the enrollment of node-negative patients, 6 articles because of the lack of a defined node-positive group, 6 articles because of the lack of available data, and 8 articles because of SLNB before NAC. Finally, 15 articles were included in this review (Figure 1).

A total of 2,471 patients in 15 studies meeting the inclusion criteria were analyzed. The 15 studies were published between 2007 and 2013. All studies exhibited an original and defined group of patients who were clinically node-positive at presentation. Five studies came from USA[12]–[14], [19], [23], and the remaining studies came from different countries. The number of sentinel lymph nodes identified and the clinically complete response to NAC in different studies ranged from 1.0 to 3.0 and 21.5% to 83.9%, respectively. Four studies did not identify clinically node-positive patients by FNA[15]–[17], [24]. Three studies only included patients who achieved clinically complete responses of axillary nodes after NAC[17], [20], [25]. With respect to the mapping technique, blue dye alone was used in one study[25], radioactive isotopes alone were used in three studies[15], [21], [22], a combination of blue dye and radionuclides was used in five studies[16], [19], [20], [24], [26], and mixed techniques were used in the other six studies[12]–[14], [17], [18], [23]. Seven studies performed additional IHC staining with anti-cytokeratin antibodies on negative nodes according to routine hematoxylin and eosin (H&E) staining[15], [16], [18], [20], [22], [25], [26]. Sentinel lymph nodes with micro-metastases (<2 mm) were considered positive in two studies[22], [25] (Table 2).

**Table 2:** Characteristics of the included studies.

Author	Years	Origin	FNA	cCR (%)	ycN- only	Mapping method	IHC	Number of Sln
Alvarado et al.[12]	2012	USA	Yes	52.7	No	4	No	2.6
Boughey et al.[13]	2013	USA	Yes	83.9	No	4	No	m
Brown et al.[14]	2010	USA	Yes	m	No	4	No	2.0
Canavese et al.[15]	2011	Italy	No	62.5	No	2	Yes	1.7
Classe et al.[16]	2009	France	No	21.5	No	3	Yes	1.9
Kuehn et al.[17]	2013	Germany	No	82.8	Yes	4	No	2.0
Lee et al.[18]	2007	Korea	Yes	21.5	No	4	Yes	m
Newman et al.[19]	2007	USA	Yes	m	No	3	No	3.0
Ozmen et al.[20]	2010	Turkey	Yes	26.0	Yes	3	Yes	2.1
Park et al.[21]	2013	Korea	Yes	40.8	No	2	No	1.0
Rebollo-Aguirre et al.[22]	2012	Spain	Yes	m	No	2	Yes	1.0
Shen et al.[23]	2007	USA	Yes	58.0	No	4	No	2.0
Takei et al.[24]	2013	Japan	No	m	No	3	No	2.9
Thomas et al.[25]	2011	India	Yes	m	Yes	1	Yes	1.6
Yagata et al.[26]	2013	Japan	Yes	m	No	3	Yes	2.0

m: missing value. Year: publication year. FNA: fine needle aspiration. cCR: clinically complete response of axillary lymph nodes to NAC. ycN-: clinically node-negative patients after NAC. Mapping method: 1 = blue dye alone, 2 = radioactive isotope alone, 3 = combination blue dye and radioactive isotope, and 4 = mix of the above-listed methods. IHC: immunohistochemistry performed on negative nodes according to H&E staining. Number of Sln =  mean number of sentinel lymph node removed.

### 3.3 Measures of test performance of SLNB

#### 3.3.1 IR of SLN

**Figure 2:**
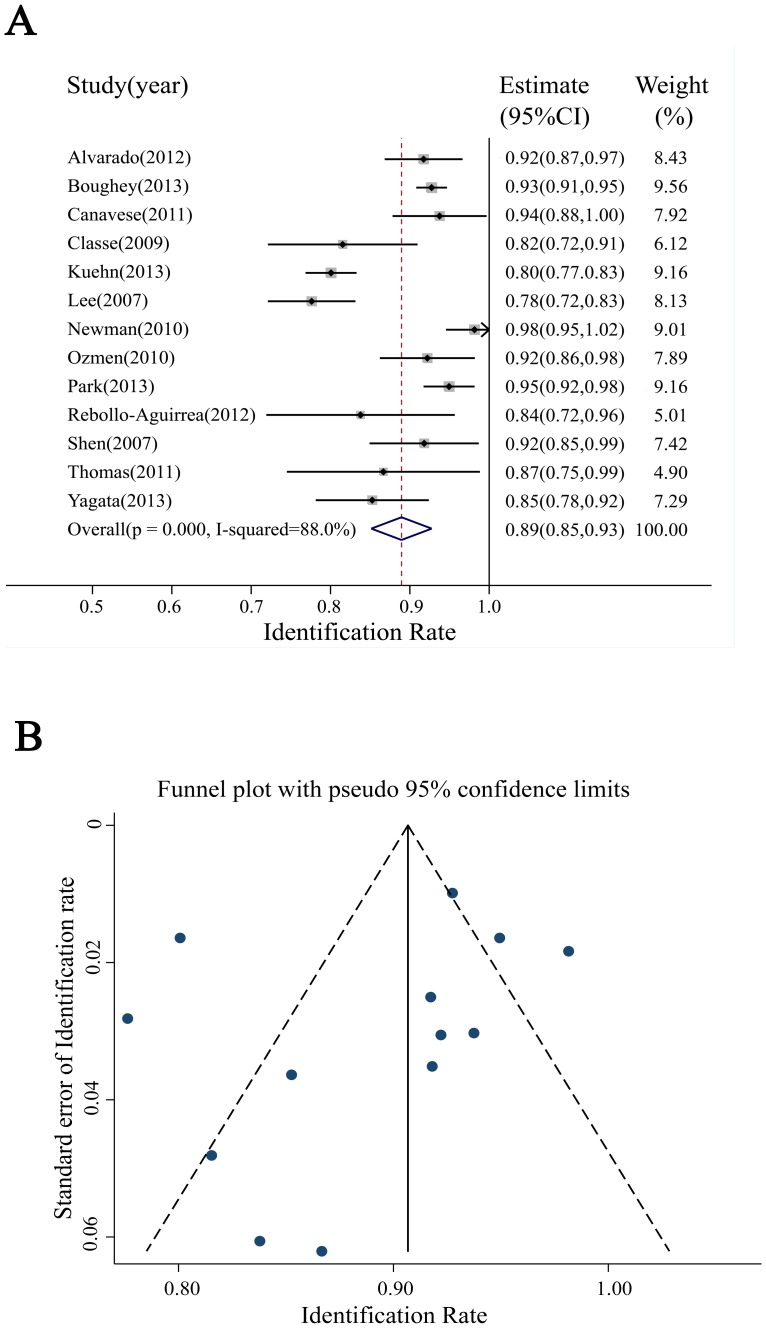
Meta-analysis of the IR. (A) Forest plot of the IR. The width of the horizontal line represents the 95% CI of individual studies. The vertical dotted line represents the overall expected IR. The combined estimate of IR was 89% (95% CI:85–93%, I^2^ = 88.0%). (B) Funnel plot to assess publication bias effect on the IR. Each dot represents a separate study. The funnel plot revealed no apparent evidence of publication bias.

Two studies provided no data to analyze IR. In the remaining 13 studies, the IR in individual studies ranged from 78% to 98%. The I^2^ was found to be 88.0%, reflecting heterogeneity of IR among the studies. Hence, a random effects model was used to estimate the combined IR, with a result of 89% [95% confidence interval (CI):85–93%] (Figure 2A). With respect to IR, funnel plots were generated to assess the publication bias of the literature, suggesting minimal bias. This result was confirmed by Begg's test, with *P* = 0.200. However, four studies were not plotted in the funnel plots (Figure 2B).

#### 3.3.2 FNR of SLNB

**Figure 3:**
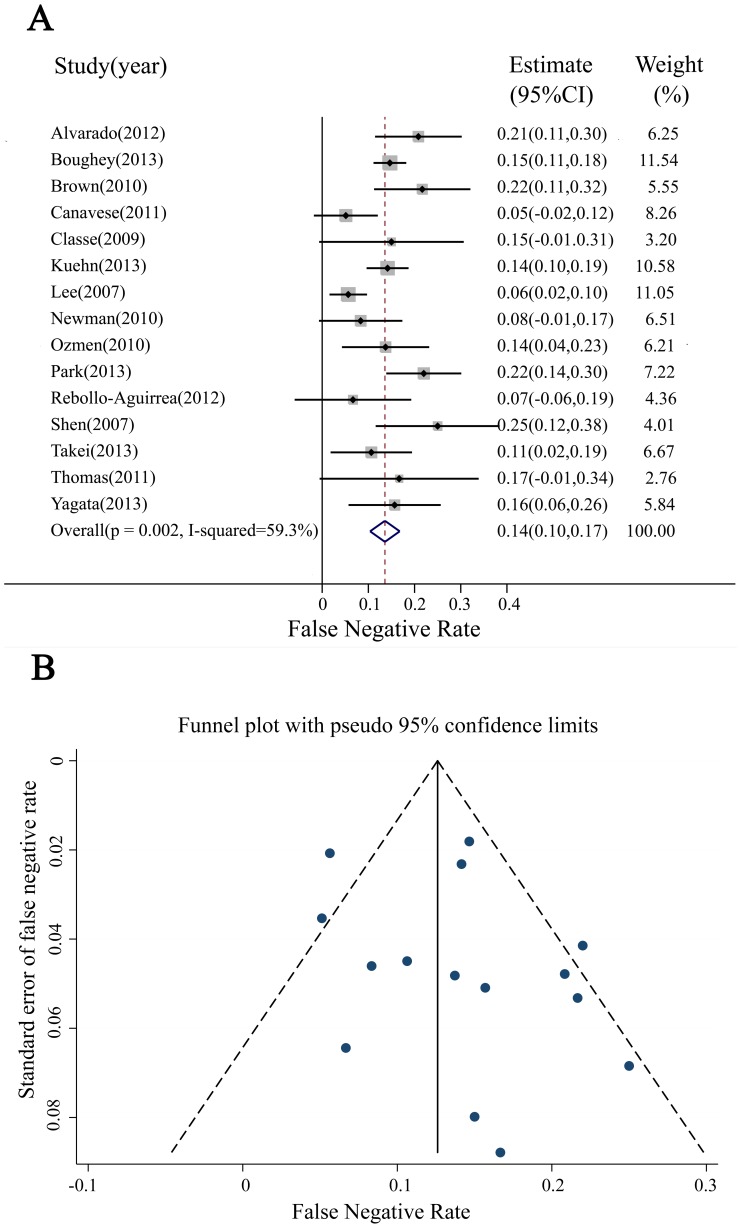
Meta-analysis of the FNR. (A) Forest plot of the FNR. The width of the horizontal line represents the 95% CI of individual studies. The vertical dotted line represents the overall expected FNR. The combined estimate of FNR was 14% (95% CI:10–17%, I^2^ = 59.3%). (B) Funnel plot to assess publication bias effect on the FNR. Each dot represents a separate study. The funnel plot revealed no apparent evidence of publication bias.

The FNR in individual studies ranged from 6% to 25% for the 15 total studies. Pooled analysis revealed that the combined FNR was 14% (95%CI 10–17%) (Figure 3A) with heterogeneity (I^2^ = 59.3%, *P* = 0.01). The funnel plot revealed minimal publication bias in terms of FNR, with three studies not plotted in the funnel plot (Figure 3B). Begg's test confirmed the above conclusions, with *P* = 0.488.

To explore the heterogeneity of FNR, meta-regression was analyzed using four variables: status of axillary lymph nodes after NAC (positive or negative); sample size (>100 cases or <100 cases); FNA (yes or no); and IHC (yes or no) (with *P* = 0.09, 0.66, 0.52, and 0.04, respectively). IHC was identified as an independent factor underlying the heterogeneity of FNR (*P* = 0.04). Stratified analysis with IHC revealed that FNR was significantly lower in the IHC plus H&E staining subgroup than in the H&E staining alone subgroup, with values of 8.7% versus 16.0% (*P* = 0.001).

#### 3.3.3 NPV and the accuracy of SLNB

The NPV in the individual studies ranged from 62% to 94%. With pooled analysis, the combined NPV was 83% (95%CI 79–88%, I^2^ = 64.1%)(Figure S1). The accuracy in individual studies ranged from 82% to 97%. With pooled analysis, the combined accuracy was 92% (95%CI 90–94%, I^2^ = 55.2%) (Figure S2) (Table 3).

**Table 3:** Test performance measurements of SLNB after NAC in individual studies.

Author	N	IR (%)	FNR (%)	NPV (%)	Accuracy (%)
Alvarado et al.[12]	121	92	21	72	86
Boughey et al.[13]	689	93	15	82	91
Brown et al.[14]	86	m	22	67	85
Canavese et al.[15]	64	94	5	91	97
Classe et al.[16]	65	82	15	92	94
Kuehn et al.[17]	592	80	14	89	93
Lee et al.[18]	219	78	6	87	96
Newman et al.[19]	54	98	8	85	94
Ozmen et al.[20]	77	92	14	74	90
Park et al.[21]	178	95	22	76	87
Rebollo-Aguirre et al.[22]	37	84	7	94	97
Shen et al.[23]	61	92	25	62	82
Takei et al.[24]	103	m	11	92	95
Thomas et al.[25]	30	87	17	73	88
Yagata et al.[26]	95	85	16	79	90
Pooled analysis	2471	89	14	83	92

m: missing value. N = number of patients. IR: identification rate. FNR: false negative rate. NPV: negative predictive value.

## Discussion

There have been three meta-analyses concerning SLNB after NAC. The first two studies included either clinically node-positive or node-negative patients prior to NAC[27], [28]. In addition, Classe and colleagues confirmed that node-positive patients prior to NAC exhibited a higher FNR than node-negative patients[16]. In 2011, the third meta-analysis indicated that for clinically node-negative patients, the FNR of 7% was similar to the FNR in patients without NAC[29]. The value of SLNB after NAC is more significant for clinically node-positive diseases, which are considered contraindications to SLNB. Once SLNB replaces ALND after positive nodes are converted to negative nodes after NAC, the number of candidates for axillary-conserving surgery will be increased. In recent years, some SLNB studies have focused on node-positive patients after NAC. The present meta-analysis aimed to evaluate SLNB in clinically node-positive patients after NAC.

### 4.1 Identification rate (IR)

The pooled analysis revealed that the IR of sentinel lymph nodes was 89% after NAC for node-positive patients. The meta-analysis of Miltenburg et al. indicated that the IR was 84%, and subgroup analysis revealed that a lower IR was related to the mapping technique[30]. In earlier years, poorer SLNB techniques may have been the main factor resulting in lower IR, as an IR of 96% was published in a subsequent meta-analysis. In addition, Xing et al.[28] and van Deurzen et al. [27] separately meta-analyzed SLNB after NAC, and both studies reported IRs of 90%. The meta-analysis of Tan et al.[29] indicated that the IR was 94% after NAC in clinically node-negative patients. In our review, the IR was 89% after NAC in clinically node-positive patients, which was comparable to the results of other meta-analyses. Therefore, SLNB after NAC is feasible for node-positive patients. However, the IR was clearly different across our included studies, indicating significant heterogeneity. In the study by Kuehn et al.[17], the factors contributing to the lower IR values were analyzed, and the results revealed that the mapping technique was an independent factor. In contrast, radioactive isotopes or combinations with blue dye produced higher IRs. Thirteen studies that were retrieved to analyze IR exhibited no uniform method; thus, it was difficult to further analyze the impact of mapping method on IR.

### 4.2 False negative rate (FNR)

The FNR of SLNB was 14% after NAC for node-positive patients, which was higher than that for node-negative patients without NAC (4–5%) [30], [31] or node-negative patients after NAC (7%)[29]. This phenomenon may be explained as follows. First, for node-positive patients, the involved lymphatic channel may be obstructed by cancer emboli or debris such that mapping agents are diverted to another uninvolved lymphatic channel. Second, the different method used for sterilization of the tumor in the lymph node after NAC will also impact the accuracy of SLNB. If the sterilization begins from the sentinel lymph node, the non-sentinel lymph node may still contain tumor cells. In light of the former explanation, management of the whole procedure in node-positive patients is proposed. In particular, clinically suspicious metastasis lymph nodes should be marked and examined as the clinical sentinel lymph node. When SLNB is performed, such clinically suspicious lymph nodes should also be removed as sentinel lymph nodes. In addition, the mapping lymph node and clinically suspicious lymph nodes should be integrated into sentinel lymph nodes. This strategy more accurately represents the entity of sentinel lymph nodes and was used in the study by Takei et al.[24] to produce an FNR of 11%.

Factors that may impact FNR were analyzed with meta-regression, in which IHC was identified as an independent factor for the heterogeneity of FNR (*P* = 0.04). Moreover, stratified analysis revealed that the FNR decreased from 16.0% to 8.7% when IHC was added to negative nodes according to H&E staining (*P* = 0.001). The meta-analysis of Tan et al. [29] also indicated that IHC could decrease the FNR after NAC in node-negative patients from 12% to 9%. In clinical practice, if sentinel lymph nodes are negative according to H&E staining, IHC should be added to achieve a more accurate outcome.

Additionally, the status of lymph nodes after NAC was considered another factor related to FNR[1717,20,25]. However, in our review, meta-regression analysis indicated that the status of lymph nodes after NAC did not contribute significantly to the heterogeneity of FNR (*P* = 0.09). The standard of conversion to negative nodes was not consistent across studies, and it was therefore difficult to determine the status of lymph nodes after NAC. Both physical examination and ultrasonic imaging were too subjective, with an inaccuracy rate of more than 30%[32]. Improvements in such detection technology will help to select more suitable subgroups of patients to receive SLNB. In addition, the impact of sample size and FNA was assessed by meta-regression analysis, and the results revealed that neither was an independent factor for the heterogeneity of FNR.

Only studies published in the English language were included, which may have led to publication bias. However, our selected studies included results from various countries. In addition, publication bias was analyzed by constructing a reverse funnel plot, and with respect to the pooled analysis of IR and FNR, the result indicated there was no publication bias. Thus, after confirmation with Begg's test, we can conclude that the results are reliable.

There were several limitations in our review. First, the IR was pooled by rate and standard error of the rate, which resulted in heterogeneity. Thus, only the random effects model could be used (as compared to the fixed effects model), and as a result, the reliability of our results was impacted. Second, the mapping technique included four categories (i.e., blue dye alone, radioactive isotope alone, combination blue dye and radioactive isotope, and all methods), and meta-regression could not be performed on these variables.

## Conclusion

SLNB is feasible after NAC for node-positive breast cancer, with an acceptable IR. However, SLNB is not sufficiently accurate to replace ALND, although IHC may improve the accuracy of SLNB.

## Supporting Information

Checklist S1 PRISMA Checklist.
(DOC)

Figure S1 Forest plot of the NPV. The width of the horizontal line represents the 95% CI of individual studies. The vertical dotted line represents the overall expected NPV. The combined estimate of NPV was 83% (95% CI: 79–87%, I^2^ = 64.1%).
(TIF)

Figure S2 Forest plot of accuracy. The width of the horizontal line represents the 95% CI of individual studies. The vertical dotted line represents the overall expected accuracy. The combined estimate of accuracy was 92% (95% CI: 90–94%, I^2^ = 55.2%).
(TIF)

File S1 Signaling questions adopted in quality assessment.
(DOCX)
