# Supplementary figures and images for: Feasibility and Accuracy of Sentinel Lymph Node Biopsy in Clinically Node-Positive Breast Cancer after Neoadjuvant Chemotherapy: A Meta-Analysis
Source: PLoS One. 2014 Sep 11;9(9):e105316. doi: 10.1371/journal.pone.0105316 (PMC4161347; doi:10.1371/journal.pone.0105316)

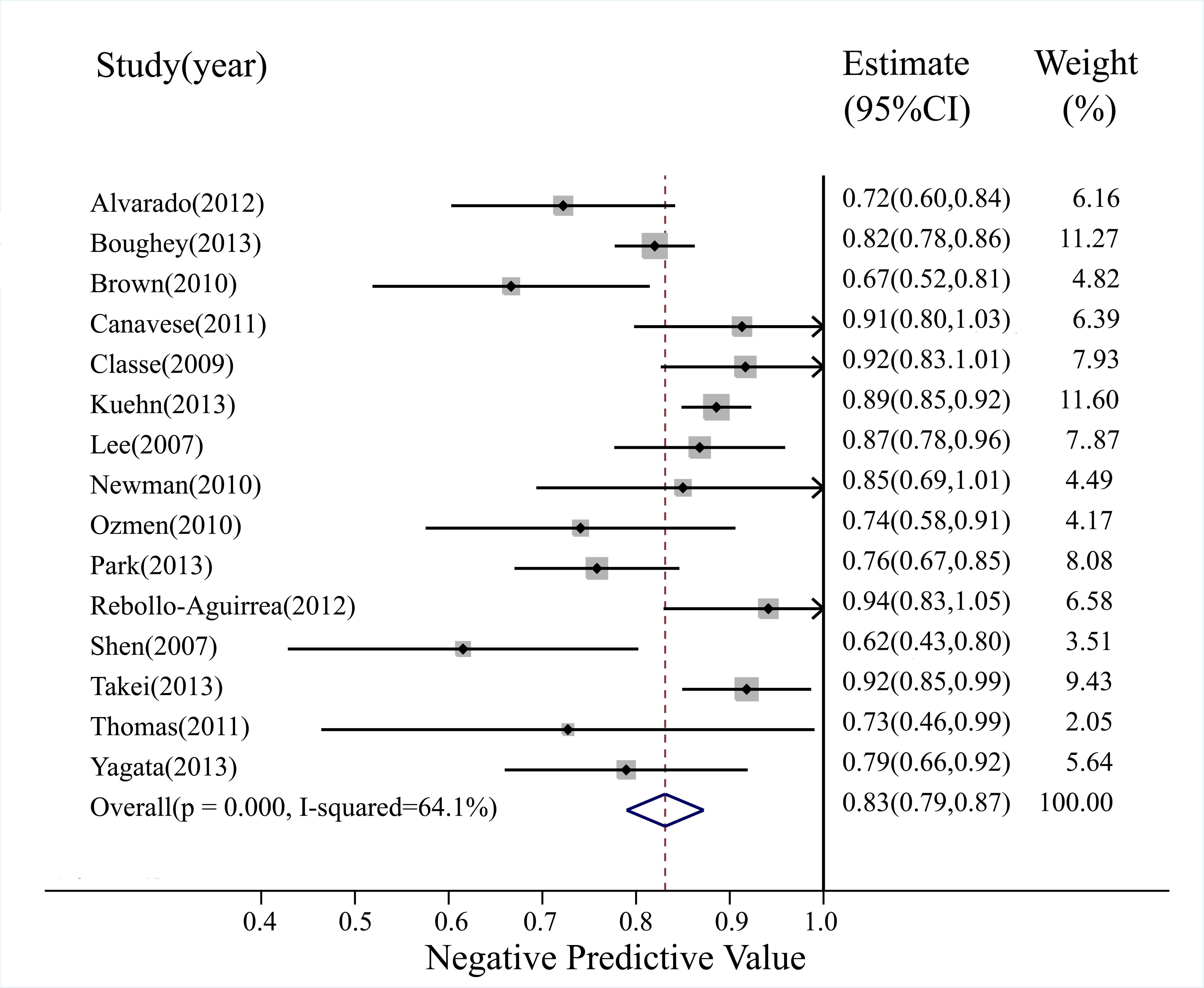

Supplement: Figure S1 — Forest plot of the NPV. The width of the horizontal line represents the 95% CI of individual studies. The vertical dotted line represents the overall expected NPV. The combined estimate of NPV was 83% (95% CI: 79–87%, I2 = 64.1%). (TIF) [file pone.0105316.s002.tif]

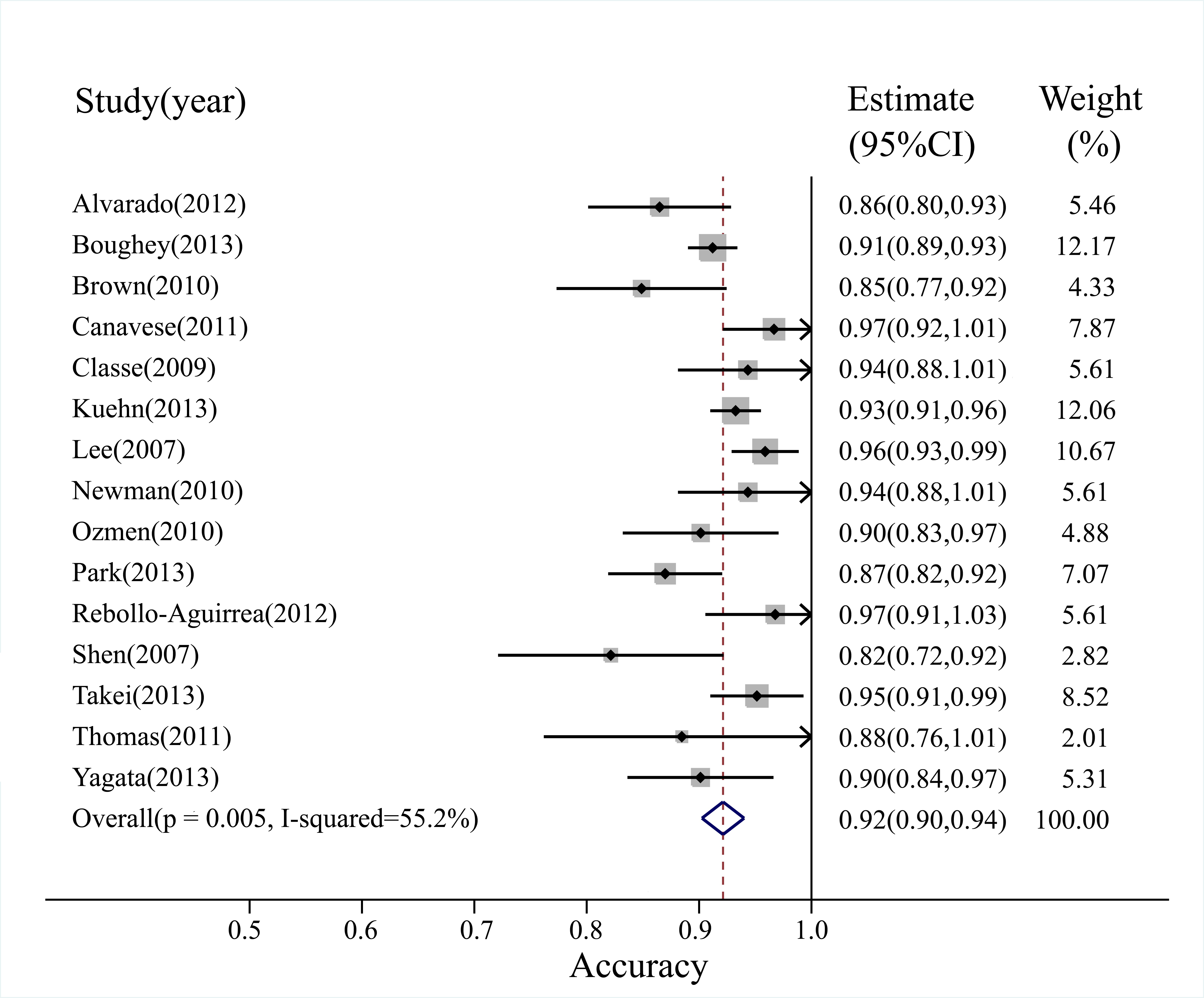

Supplement: Figure S2 — Forest plot of accuracy. The width of the horizontal line represents the 95% CI of individual studies. The vertical dotted line represents the overall expected accuracy. The combined estimate of accuracy was 92% (95% CI: 90–94%, I2 = 55.2%). (TIF) [file pone.0105316.s003.tif]
